# Brain-Specific Oxysterols and Risk of Schizophrenia in Clinical High-Risk Subjects and Patients With Schizophrenia

**DOI:** 10.3389/fpsyt.2021.711734

**Published:** 2021-08-02

**Authors:** Zuoli Sun, Lei Zhao, Qijing Bo, Zhen Mao, Yi He, Tao Jiang, Yuhong Li, Chuanyue Wang, Rena Li

**Affiliations:** ^1^The National Clinical Research Center for Mental Disorders and Beijing Key Laboratory of Mental Disorders, Beijing Anding Hospital, Capital Medical University, Beijing, China; ^2^Beijing Institute for Brain Disorders, Capital Medical University, Beijing, China; ^3^Advanced Innovation Center for Human Brain Protection, Capital Medical University, Beijing, China

**Keywords:** schizophrenia, cholesterol metabolism, biomarker, 24(S)- hydroxycholesterol, 27-hydroxycholesterol

## Abstract

Accumulating evidence from clinical, genetic, and epidemiologic studies suggest that schizophrenia might be a neuronal development disorder. While oxysterols are important factors in neurodevelopment, it is unknown whether oxysterols might be involved in development of schizophrenia. The present study investigated the relationship between tissue-specifically originated oxysterols and risk of schizophrenia. A total of 216 individuals were recruited in this study, including 76 schizophrenia patients, 39 clinical high-risk (CHR) subjects, and 101 healthy controls (HC). We investigated the circulating levels of brain-specific oxysterol 24(S)-hydroxycholesterol (24OHC) and peripheral oxysterol 27-hydroxycholesterol (27OHC) in all participants and analyzed the potential links between the oxysterols and specific clinical symptoms in schizophrenic patients and CHR. Our data showed an elevation of 24OHC in both schizophrenia patients and CHR than that in HC, while a lower level of 27OHC in the schizophrenia group only. The ratio of 24OHC to 27OHC was only increased in the schizophrenic group compared with CHR and HC. For the schizophrenic patients, the circulating 24OHC levels are significantly associated with disease duration, positively correlated with the positive and negative syndrome total scores, while the 27OHC levels were inversely correlated with the positive symptom scores. Together, our data demonstrated the disruption of tissue-specifically originated cholesterol metabolism in schizophrenia and CHR, suggesting the circulating 24OHC or 24OHC/27OHC ratio might not only be a potential indicator for risk for schizophrenia but also be biomarkers for functional abnormalities in neuropathology of schizophrenia.

## Introduction

Schizophrenia is a chronic mental disorder characterized by delusions, hallucinations, impaired cognition, behavior, or emotions. While the actual causes of schizophrenia are not fully understood, recent studies support the neurodevelopmental hypothesis of schizophrenia (1). Cholesterol is well-known for its roles in brain development, such as proper myelination, dendritic differentiation, and synaptic plasticity in central nervous system (CNS) (2–4). While circulating cholesterol is not able to cross the blood-brain barrier (BBB) into the brain, brain synthesize cholesterol locally for cell maintenance, neuronal transmission, and synapse formation. Studies have shown that maining cholesterol homeostasis is essential for proper cellular and systemic functions, while disturbance cholesterol homeostasis might increase risks for many neurodevelopmental disorders (5–7). Although schizophrenia is a developmental disease and alteration of circulating cholesterol levels were reported in schizophrenic patients (8–12), it is unclear whether brain and peripheral cholesterol metabolism are disturbanced in schizophrenia and what their relationship related to this disease.

Oxysterols, the oxidized forms of cholesterol are able to cross BBB from peripheral to brain and vice versa (13). 24(S)-Hydroxycholesterol (24OHC), for example, is the major cholesterol metabolite in the brain by brain-specific enzyme CYP46A1. The plasma level of 24OHC is considered a surrogate marker for brain cholesterol metabolism since more than 90% of plasma 24OHC can be attributed to the brain (14, 15). It has been confirmed that the level of circulating 24OHC derived from the brain (16) is positively correlated with the 24OHC level in the CSF (17). In contrast to the brain-specifically originated 24OHC, 27-hydroxycholesterol (27OHC) as the most abundant oxysterol in the circulating system is the product of 27-hydroxylase (CYP27A1), mainly formed from peripheral as extrahepatic and extracerebral cholesterol (13).

Cumulating evidence indicates the positive role of 24OHC on dendritic spines, synaptic plasticity, and synaptic vesicle cycling (18–20). As an allosteric modulator of the NMDA receptors, 24OHC also increases synaptic plasticity in hippocampal slices (21, 22). Due to the important role of oxysterols during neurodevelopment and cognition, many studies indicated the impact of oxysterol disturbance in brain disorders, such as Alzheimer's disease (23–25), amyotrophic lateral sclerosis (26), Parkinson's disease (27), major depressive disorder (MDD) (28), and autism spectrum disorder (ASD) (29). Although accumulating evidence indicates the lipid metabolism, such as eicosanoid signaling were associated with the pathophysiology of schizophrenia (30, 31), cholesterol metabolism and oxysterols in schizophrenia is not well-studied. A recent study reported no significant difference in plasma 24OHC levels between schizophrenic patients and healthy controls (32), and one study used 24OHC-liked compound reversed schizophrenia-liked behavioral in animal models and suggested a potential therapeutic effect of 24OHC on schizophrenia (22). As the most abundant oxysterol in the circulating system, 27OHC levels increased when hypercholesteremia or oxidative stress (33, 34), and then as the mediator of the negative effects on cholesterol metabolism and cognitive function by decreasing the expression of HMG-CoA reducatase (the rate limit of cholesterol synthesis) (35). Plasma levels of 27OHC were proved to be associated with cognitive impairment patients, including mild cognitive impairment (MCI) and AD (36–38) due to its deleterious impact to synaptic plasticity (39–41). However, 27OHC has not been investigated previously in schizophrenia.

Given the different origins of the two oxysterols and circulating 24OHC and 27OHC could represent brain and peripheral cholesterol metabolites, we first investigated whether the two tissue-specific oxysterols were associated with risk for schizophrenia by comparing the circulating 24OHC and 27OHC levels as well as cognitive function in schizophrenic patients, CHR compared with HC. Then, we studied the impact of oxysterols in specific psychiatric symptoms in schizophrenia patients and CHR subjects. Our study suggested that tissue-specifically originated oxysterols might be valuable targets for early intervention and alternative treatment for schizophrenia.

## Materials and Methods

### Subjects

This study was reviewed and ratified by the Independent Ethics Committee (IEC) of the Beijing Anding Hospital, Capital Medical University, China. Each subject provided informed written consent after the procedure had been fully explained in the present study.

Diagnosis of schizophrenia was confirmed by administering the Structured Clinical Interview for DSM-IV (SCID) by experienced psychiatrists. CHR individuals met diagnostic criteria for a psychosis-risk syndrome, the Criteria of Psychosis-Risk States based on the face-to-face interview using Structured Interview for Psychosis-Risk Syndromes (SIPS) (42). Healthy controls (HCs) did not meet criteria for any prodromal syndrome, had any history of psychiatric illness or psychoactive drug use, or had no first-degree relative with mental disorders.

Participants in each group were excluded if they (1) aged under 16 or above 60; (2) had other neurological disorder; (3) had a history of drug or alcohol abuse; (4) were pregnancy or currently breastfeeding; and (5) were in significant medical conditions, including severe cardiovascular and hepatic or renal diseases.

Total of 216 participants, including 76 schizophrenia patients, 39 CHR subjects, and 101 age- and sex-matched HCs, were enrolled in this study from Beijing Anding Hospital, China (Table 1). The period from psychotic symptom appeared for the first time to the enrollment (psychotic symptom duration) was 23.79 months (SD = 24.05) in schizophrenia patients and 25.50 months (SD = 28.35) in CHR participants. Medication information was unavailable for six patients and five individuals in CHR group. For the patients who had medication information, 53 patients were not taking antipsychotics at the time of enrollment, while 17 patients taking antipsychotics, including risperidone, clozapine, olanzapine, quetiapine, paliperidone, and amisulpride. For the CHR subjects who had medication information, 24 subjects were not taking antipsychotics at the time of enrollment, while 10 subjects taking antipsychotics.

**Table 1:** Demographic variables and clinical characteristics in schizophrenia patients, CHR individuals, and HCs.

	**HC**	**CHR**	**SCZ**	**F**	***P***
***N***	**101**	**39**	**76**		
Age (year)	26.45 ± 4.57	24.28 ± 4.78	25.68 ± 6.85	2.185	0.115
Gender (M/F)	62/39	24/15	34/42	5.559	0.062
Education (year)	14.35 ± 3.18	14.39 ± 3.02	13.37 ± 3.27	2.202	0.113
BMI	22.56 ± 2.95	23.11 ± 2.82	22.97 ± 3.34	0.369	0.692
Family history (yes/no)	0/101	11/28	15/57	27.773	<0.001
Illness/psychotic symptom duration (month)	–	25.50 ± 28.35	23.79 ± 24.05	0.103	0.740
Antipsychotics (yes/no)	–	10/24	17/53	–	–
Olanzepine equivalents (mg)	–	14.00 ± 7.75	8.54 ± 4.14	–	–
**Cognition**					
Information processing speed	44.56 ± 6.88	39.03 ± 6.78	36.93 ± 19.43	4.886	0.015
Attention alertness	45.54 ± 9.09	39.75 ± 11.85	31.43 ± 9.17	26.072	<0.001
Working memory	46.33 ± 6.88	35.02 ± 17.14	37.14 ± 10.43	12.435	<0.001
Vocabulary learning	47.51 ± 9.44	41.77 ± 9.37	38.49 ± 9.87	11.910	<0.001
Visual learning	46.34 ± 10.34	42.67 ± 11.03	38.38 ± 14.48	5.894	0.007
Reasoning and problem solving	43.32 ± 10.52	39.62 ± 10.54	35.87 ± 11.40	6.768	0.004
Social cognition	37.79 ± 10.43	37.03 ± 8.68	34.85 ± 12.46	1.475	0.399
Total MCCB scores	44.40 ± 5.88	40.21 ± 5.62	35.88 ± 6.36	24.826	<0.001
**Psychiatric symptoms**					
SIPs	0.76 ± 2.69	25.41 ± 8.63	–	365.055	<0.001
PANSS positive	–	–	23.07 ± 5.58	–	–
PANSS negative	–	–	20.41 ± 8.02	–	–
PANSS general	–	–	41.88 ± 6.48	–	–
PANSS total	–	–	85.37 ± 14.17	–	–
**Oxysterols**					
24OHC (ng/ml)	24.47 ± 8.76	28.54 ± 12.18	27.89 ± 8.95	3.990	0.020
27OHC (ng/ml)	43.14 ± 18.30	43.96 ± 14.63	32.78 ± 11.92	11.296	<0.001

*Data was shown as mean ± SD*.

*SCZ, schizophrenia; CHR, clinically high risk; HC, healthy control; BMI, body mass index; IQ, intelligence quotient; MCCB, MATRICS consensus cognitive battery; SIPS, structured interview for prodromal symptoms; CGI, clinical global impressions severity; PANSS, Positive and Negative Syndrome Scale; 24OHC, 24(S)-hydroxycholesterol; 27OHC, 27-hydroxycholesterol*.

### Clinical Assessments

Basic sociodemographic characteristics and clinical data were collected by a questionnaire specifically designed for this study. All schizophrenia patients were assessed using the Positive and Negative Syndrome Scale (PANSS) as a further assessment of the disease (43). The SIPS which includes 19 items, divided into positive, negative, disorganization, and general symptom subsections, was used for identifying the state of the CHR individuals (42).

The cognitive function was assessed with MATRICS Consensus Cognitive Battery (MCCB, Chinese version) (44), which provides a comprehensive score to act as a cognitive reference point for schizophrenia individuals. In addition, the intelligence quotient (IQ) was evaluated by a Chinese version of the Wechsler Adult Intelligence Scale (45). Although all subjects completed the clinical assessments, a total of 138 subjects (50 HCs, 38 CHR individuals, and 50 schizophrenia patients) completed the cognitive evaluation.

### Oxysterol Analysis

The whole blood of subjects was collected into EDTA tubes after clinical assessment at 8:00 a.m. to 15:00 p.m. Plasma was harvested after centrifugation at 3,000 rpm for 10 min at room temperature. Plasma levels of oxysterols were measured using high-performance liquid chromatography**-**mass spectrometry (HPLC**-**MS) as described previously with modifications (46). Briefly, a total of 50 μl plasma, 100 ng of D5/D7 deuterium cholesterol, and 200 μl acidic buffer solution (50 mM ammonium acetate, 1% formic acid, pH = 3) were mixed with 1 ml methyl tert butyl ether in an eppendorf tube. Supernatant was gathered after the freezing at −80°C and then dried at 30°C. Fifty microliters of chloroform solution with 12.6 g/L *N,N*′-diisopropylcarbodiimide, 12.4 g/L nicotinamide, and 12.2 g/L 4-dimethylaminopyridine was added to derive at 35°C water bath for 2 h. The reactants were dried and dissolved in 100 μl of methanol for HPLC-MS detection. HPLC with an Angilent G1312B HPLC Pump and an Angilent C18 column (0.35 μm bead size; 4.6 × 250 mm) were used for the measurement of oxysterols.

### Statistical Analysis

The data was analyzed with SPSS (version 20.0, SPSS Inc., Chicago, Illinois, USA). Descriptive statistics were used to describe demographic and clinical characteristics of the participants. The variables were expressed as mean ± standard deviation (SD). Comparisons of demographic, clinical variables, and oxysterol levels among groups were performed using one-way analysis of variance (ANOVA) followed by *post-hoc* Bonferroni multiple comparison test, while gender and family history were performed using chi-squared test. A multiple linear regression analysis was used to assess the factors affecting oxysterol levels in schizophrenia patients, including age, gender, illness duration, antipsychotics, and family history. Spearman's correlation analysis was used to evaluate the relationship between plasma oxysterol levels and clinical parameters or age in subjects. Multiple linear regression analysis was used to assess the relationship between oxysterols and demographic variables or clinical characteristics in schizophrenia patients. The receiver operating characteristic (ROC) curve and the area under the ROC curve (AUC) were used to evaluate the diagnosis value of oxysterols in schizophrenia. The level of two-tailed statistical significance was also set to *p* < 0.05 for all tests.

## Results

Table 1 summarizes the demographic, psychiatric symptoms, and cognitive assessments of the participants.

### Opposite Changes Between Brain and Peripheral Cholesterol Metabolites in Participants

**Figure 1:**
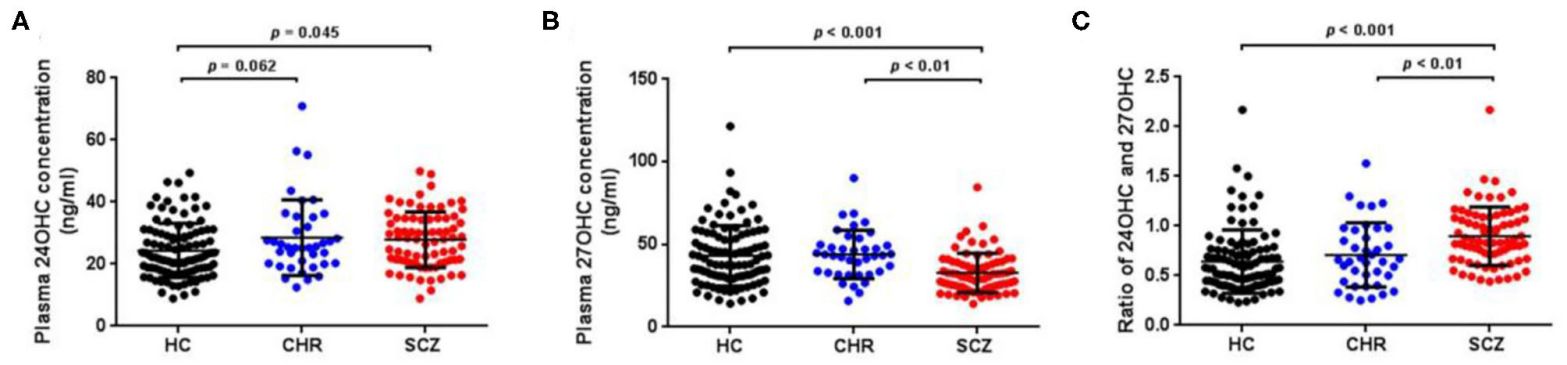
Plasma oxysterols profile in schizophrenia (SCZ) patients, clinically high risk (CHR) individuals, and healthy controls (HC). The plasma 24OHC levels, 27OHC levels, and 24OHC/27OHC in SCZ patients, CHR, and HC are shown in **(A)**, **(B)**, and **(C)**, respectively. SCZ patients showed higher plasma 24OHC levels and lower 27OHC levels than HCs.

Mean plasma 24OHC levels revealed a significant increase in schizophrenia patients (Table 1; Figure 1A, *p* = 0.045) and an increase trend in CHR participants (*p* = 0.062) relative to HCs. On the other hand, schizophrenia patients showed significantly lower plasma 27OHC levels than HCs (Figure 1B, *p* < 0.001), however, no such change was found in the CHR group.

A multiple linear regression analysis was used to assess the factors affecting oxysterol levels in schizophrenia patients, including age, gender, illness duration, antipsychotics, and family history. Significant correlations were found between 24OHC and age (*t* = −3.141, *p* = 0.003) or illness duration (*t* = 3.023, *p* = 0.004). However, sex, antipsychotics and family history had no significant influence on oxysterol levels (all *p* > 0.05).

### Changes in Age-Dependent Oxysterol Levels in Schizophrenia Patients and CHR Individuals Compared With HCs

**Figure 2:**
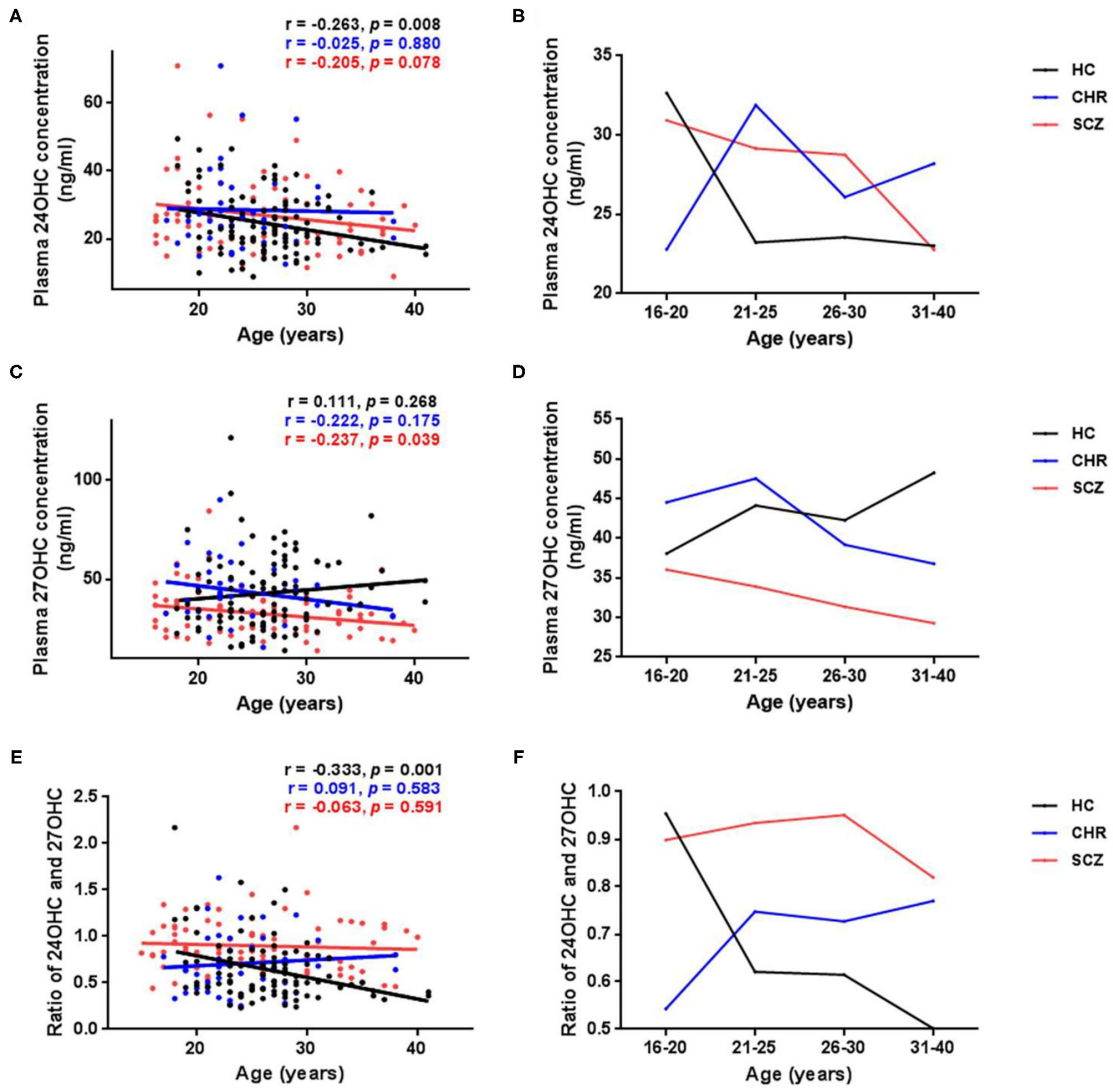
Age-related changes of plasma levels of oxysterols in schizophrenia (SCZ) patients, clinically high risk (CHR) individuals, and healthy controls (HC). **(A,B)** Absolute values of 24OHC at different ages; **(C,D)** Absolute values of 27OHC at different ages. **(E,F)** Ratio of 24OHC and 27OHC at different ages. Significant age-dependent decreases of 24OHC levels and 24OHC/27OHC values were found in the HCs. Opposite trend of 24OHC levels and 24OHC/27OHC values along with age were found between HCs and CHR individuals or SCZ patients, and the largest difference occurs before 25 years old. Plasma 27OHC levels showed significant age-dependent decrease in the SCZ group. Unlike 24OHC, the difference in 27OHC between patients and HCs became more and more significant with the increase of age.

A significant age-dependent decrease in 24OHC levels was found in the HCs (Figure 2A, *p* = 0.008); however, similar correlation disappeared in CHR (*p* = 0.880) or schizophrenia patients (*p* = 0.078). Interestingly, the mean 24OHC levels in different ages showed obvious change in CHR compared with HCs, and the largest difference was found in the subjects before 25 years old among groups (Figure 2B). On the other hand, the plasma 27OHC levels showed significant age-dependent decrease in schizophrenia patients (Figure 2C, *p* = 0.039) or unchanged in CHR individuals (*p* = 0.175), although slow age-dependent increase was found in HCs (*p* = 0.268). Unlike 24OHC, the difference in 27OHC levels among groups became more and more significant along with age (Figure 2D). Similar to 24OHC, 24OHC/27OHC values were significantly age-dependent decreased in the HCs (Figure 2E, *p* = 0.001); however, such correlation disappeared in CHR (*p* = 0.583) or schizophrenia patients (*p* = 0.591).

### Associations of Oxysterol Levels and Cognitive Function in Each Group

Significant positive correlations between plasma 27OHC levels and MCCB total scores was found in HCs (Table 2, *p* = 0.025); however, such correlation disappeared in CHR or schizophrenia patients. No significant correlation was found between plasma 24OHC levels and cognitive function.

**Table 2:** Associations between oxysterols and clinical assessments in schizophrenia patients, CHR individuals, and HCs.

	**HC**		**CHR**		**SCZ**	
	**24OHC**	**27OHC**	**24OHC**	**27OHC**	**24OHC**	**27OHC**
Total MCCB scores	0.210 (0.186)	0.025 (0.327)	0.335 (0.193)	0.399 (−0.169)	0.077 (−0.279)	0.778 (0.045)
PANSS positive	–	–	–	–	0.806 (−0.029)	0.010 (−0.299)
PANSS negative	–	–	–	–	0.029 (0.255)	0.621 (0.059)
PANSS general	–	–	–	–	0.053 (0.228)	0.587 (0.065)
PANSS total	–	–	–	–	0.037 (0.244)	0.659 (−0.053)

*Data was shown as p-value (correlation coefficient)*.

*SCZ, schizophrenia; CHR, clinically high risk; HC, healthy control; BMI, body mass index; IQ, intelligence quotient; MCCB, MATRICS consensus cognitive battery; SIPS, structured interview for prodromal symptoms; PANSS, Positive and Negative Syndrome Scale; 24OHC, 24(S)-hydroxycholesterol; 27OHC, 27-hydroxycholesterol*.

### Associations of Oxysterol Levels and Psychiatric Symptoms in Schizophrenia Patients

Plasma 24OHC levels increased along with the PANSS total scores in the schizophrenia patients (Table 2, *p* = 0.037), especially negative symptom scores (*p* = 0.029). On the contrary, plasma 27OHC levels inversely correlated with positive symptom scores in schizophrenia patients (*p* = 0.010), although there was no significant correlation with PANSS total scores.

In order to eliminate the influence of confounding factors on psychiatric symptoms, the multiple linear regression analysis was used in this study (Table 3). There was a significant association between plasma 27OHC levels and positive symptom scores (*p* = 0.024) in schizophrenia patients. A significant correlation between 24OHC levels and PANSS total scores was found (*p* = 0.041) in schizophrenia patients.

**Table 3:** Associations between psychiatric symptoms and oxysterols levels in schizophrenia patients.

	**PANSS positive**		**PANSS negative**		**PANSS general**		**PANSS total**	
	***t***	***P***	***t***	***P***	***t***	***P***	***t***	***P***
**CHR INDIVIDUALS**								
Age	−1.158	0.260	0.290	0.775	−1.147	0.264	−0.473	0.642
Gender	−0.873	0.393	−1.018	0.320	0.725	0.476	−0.331	0.744
Illness duration	−0.617	0.544	0.245	0.809	−1.104	0.282	−0.219	0.829
Antipsychotics	0.858	0.401	−0.417	0.681	0.345	0.735	0.054	0.958
Family history	−0.985	0.336	0.039	0.970	−0.198	0.845	−0.193	0.849
24OHC	0.740	0.468	0.314	0.757	−0.223	0.826	−0.183	0.857
27OHC	−2.014	0.057	−1.369	0.186	−0.426	0.675	−0.845	0.410
**SCZ PATIENTS**								
Age	−1.816	0.074	0.080	0.936	−0.757	0.452	−0.961	0.340
Gender	3.046	0.003	−0.262	0.794	1.510	0.136	1.649	0.104
Illness duration	−0.665	0.509	0.251	0.803	−0.517	0.607	−0.329	0.743
Antipsychotics	−1.536	0.130	0.068	0.946	0.794	0.430	−0.125	0.901
Family history	−0.477	0.635	−1.532	0.131	−0.921	0.361	−1.564	0.123
24OHC	0.505	0.615	1.976	0.053	1.407	0.164	2.084	0.041
27OHC	−2.310	0.024	0.904	0.370	−0.301	0.764	−1.531	0.131

*SCZ, schizophrenia; CHR, clinically high risk; PANSS, Positive and Negative Syndrome Scale; 24OHC, 24(S)-hydroxycholesterol; 27OHC, 27-hydroxycholesterol*.

### Diagnosis Capacity of Oxysterols in Schizophrenia

We subsequently performed a ROC curve analysis to assess whether plasma oxysterol levels could differentiate the CHR individuals and schizophrenia patients from HCs (Table 4). The AUC of plasma 24OHC and 27OHC diagnosis of schizophrenia patients from the HCs was 0.618 and 0.681, respectively, and the combination of 24OHC and 27OHC was 0.767. On the other hand, the AUC of plasma 24OHC and 27OHC diagnosis of CHR individuals from the HCs was 0.591 and 0.537, respectively, and the combination of 24OHC and 27OHC was 0.592.

**Table 4:** ROC curve analysis of oxysterols in CHR participants and schizophrenia patients.

	**AUC**	**Sensitivity**	**Specificity**	**95% CI**
**CHR individuals**				
24OHC	0.591	0.525	0.692	0.488–0.695
27OHC	0.537	0.505	0.667	0.436–0.638
24OHC and 27OHC	0.592	0.525	0.692	0.488–0.696
**SCZ patients**				
24OHC	0.618	0.683	0.526	0.534–0.701
27OHC	0.681	0.485	0.842	0.603–0.759
24OHC and 27OHC	0.767	0.782	0.671	0.697–0.837

*SCZ, schizophrenia; CHR, clinically high risk; ROC, receiver operating characteristic; AUC, area under the ROC curve; CI, confidence interval; 24OHC, 24(S)-hydroxycholesterol; 27OHC, 27-hydroxycholesterol*.

## Discussion

The present study investigated the potential links between plasma levels of tissue-specific cholesterol metabolites and risks of schizophrenia, particularly in schizophrenic patients, CHR subjects, and HCs. First, we found a significant elevation in plasma 24OHC levels of schizophrenia patients as well as CHR compared with HCs (Figure 1), although the level of 24OHC in CHR group compared with HC did not reach statistical significance. As the most metabolite of cholesterol in brain, large efforts have been made in the previous to unravel the effects of 24OHC on the brain function. *CYP46A1*^–/–^ mice showed strong neurological phenotypes in spatial learning and memory deficits, indicating the essential role of 24OHC in the neuronal function (47). 24OHC has been shown to facilitate the induction of long-term potentiation (LTP) *via* enhancing NMDA (21, 22, 48) and tyrosine kinase receptor B (TrkB) signaling (19, 49). Moreover, 24OHC has a positive regulating effect on dendritic spines, enhances synaptic vesicle cycling, and increases the expression of synaptic proteins in synaptosome (18, 20). These results implied that increasing 24OHC levels to facilitate the synaptic transmission might be a therapeutic way for schizophrenia, due to the impairment of synaptic function was the main characteristic of schizophrenia (50). The 24OHC-liked compound reversed schizophrenia-liked behavioral in animal models provides strong evidence for this hypothesis (22). On the other hand, previous studies have been indicated that 24OHC may facilitate inflammation, oxidative stress, autophagy, and necroptosis, which are also main driving forces in schizophrenia (51–53). These data implied that lowering 24OHC levels might be a therapeutic way in schizophrenia treatment. Inhibition of CYP46A1 has therapeutic relevance to CNS hyperexcitability supported the therapeutic way of lowering 24OHC to schizophrenia (54). Hence, the role of 24OHC is still somewhat puzzling, but a recent report shed a new light that esterification of 24OHC is a prerequisite for inducing cell death (55). In the present study, our finding on 24OHC only increased in patients, not enough in CHR, indicating potential risk for schizophrenia. It was noteworthy that whether 24OHC is an early marker of schizophrenia still needs follow-up study of CHR, due to the conversion rate of CHR to schizophrenia within 2 years was 29.1% in Chinese sample (56).

To examine the changes of peripheral oxysterol in schizophrenia, we detected the plasma level of 27OHC in this study. Our results showed a significant reduction of 27OHC level in schizophrenia patients than HCs (Figure 1), suggesting a disruption of peripheral cholesterol metabolism in schizophrenia. As the most abundant oxysterol in the circulating system, substantial evidence indicates the facilitated effect of 27OHC on inflammation (57, 58), oxidative stress (59), immune suppressive (60), gut microbiota dysbiosis, and intestinal barrier dysfunction (61), all of which were proved to participate in schizophrenia (62–64). On the other hand, 27OHC is able to pass through the BBB and may thus contribute to the abnormal brain function. 27OHC not only affects brain function by negatively regulating cholesterol level but also directly had neurotoxic effects on neurite outgrowth and synaptic plasticity (39–41, 65, 66). Due to the strong correlation between oxysterols and plasma total cholesterol levels (67), the reduced 27OHC levels in schizophrenia patients in this study might be caused by the decrease of circulating cholesterol concentration, or the increase of degradation to bile acid. In line with our results, lower circulating cholesterol levels in schizophrenia patients than HCs were found in several studies (9, 68–70).

Activation of the liver X receptors (LXRs) was an essential pathway to exert their function of oxystrols, including 24OHC and 27OHC (71). As the ligands of LXRs, oxysterols could induce cholesterol-related genes expression to regulate the cholesterol transport and elimination. Compared with other oxysterols, 24OHC was proved to be the most efficient ligand of LXRs (72). Consideration of the opposite changes of the two kinds of oxysterols in schizophrenia patients in our study, one possibility is that elevation of 24OHC levels might induce the excessive activation of LXRs, while the reduction of 27OHC levels might not be sufficient to compensate it. It is noteworthy that a significant elevated ratio of 24OHC and 27OHC was found in schizophrenia patients in our study, suggesting increased brain/peripheral cholesterol metabolism may be a risk factor for schizophrenia. The shift of balance in 24OHC and 27OHC might cause abnormal cholesterol homeostasis and a series of consequences, such as oxidative stress and inflammation (71, 73).

As previous studies demonstrated that the level of oxysterol changes with age, (17, 74), we analyzed the age-dependent changes in all subjects and found a significant age-dependent decline of 24OHC in HC (Figure 2), not in CHR and schizophrenia groups (Figure 2). Our finding which in line with previous reports (17, 32, 74) suggested an impaired age-dependent brain-specific cholesterol turnover in CHR and schizophrenia such as the dynamic ratio of brain (synthesis of 24OHC) and liver (degradation of 24OHC) volumes (74). Similar to 24OHC, as shown in Figure 2, our study showed a different age-dependent change of 27OHC levels, such as a decline in schizophrenia patients and CHR individuals compared with a slow increase in HCs with aging as previously reported (75–77). These data suggest the disturbance of both 24OHC and 27OHC occurring in the prodromal stage of schizophrenia.

Cognitive impairment is one of the major symptoms of schizophrenia, and it is known that oxysterols might affect the cognitive function (24, 78–81). In this study, we also found 27OHC showed a significant positive association with cognitive function in HCs (Table 2). The link between oxysterols and cognitive function is confirmed in animal studies, such as both *CYP46A1* and *CYP27A1* knockout mice showed significant impairment in learning and memory (47, 78, 82). Extensive evidence showed that the oxysterols regulated cognitive function through several pathways, such as TrkB signaling pathway (18), NMDA receptors (21, 22), and nitric oxide signaling (20) to improve synaptic growth and plasticity. However, such positive correlation between oxysterols and cognition was disappeared in schizophrenia patients (Table 2). The possible reason might include the impairment of the signaling pathway which improving synaptic function in schizophrenia (83, 84). Whether the disturbed brain and peripheral cholesterol metabolites are the primary or secondary factors in schizophrenia require further investigations.

As the ratio of 24OHC to 27OHC is only upregulated in the schizophrenia group compared with the CHR and HC groups, we then investigated the association between tissue-specific oxysterols and clinical symptoms in the schizophrenia patients. First, we found that the elevated circulating level of 24OHC was associated with illness duration in the schizophrenic patients (Table 2). This finding suggested two possibilities at least. One is the increase of brain cholesterol metabolic might be correlated with disease progresses in schizophrenia, and another is the deteriorations in peripheral cholesterol homeostasis with the disease duration might be related to antipsychotic treatment in schizophrenia patients (12, 85, 86). It is believed that most cholesterol (70%) in the brain is stored in myelin and when myelin or brain cell membrane is damaged, more 24OHC will be formed from cholesterol (87, 88). Indeed, the breakdown of myelin and white matter have been reported in the schizophrenia patients with a long duration of illness (89), while others also found impaired white matter at early stage of schizophrenia (90–92). These reports support our first hypothesis that the elevated 24OHC may be related to the progressive pathophysiology, such as abnormalities of myelin in schizophrenia. Interestingly enough, similar changes of plasma 24OHC levels were also reported in ASD and MDD which shared the similar myelin or synaptic function defect as schizophrenia (28, 29, 93–96). All together prompt a critical role of brain-specific originated oxysterol in neuropathology of psychiatric disorders.

To see whether antipsychotic treatment might be involved in the changes of oxysterol levels in schizophrenia patients, we analyzed the association between oxysterols and treatments. We found no significant differences in 27OHC levels in schizophrenia patients who treated with and without antipsychotics (Supplementary Figure 1, *p* = 0.905). Our data suggest no significant impact of antipsychotics in plasma 27OHC levels. Similarly, we found no evidence of antipsychotics effected on plasma 24OHC levels in schizophrenia patients (Supplementary Figure 1, *p* = 0.995), which was consistent with previous report [Chiappelli et al., (32)]. These results suggest that the association between elevated circulating 24OHC levels with illness duration in the schizophrenic patients is more likely due to the neuropathological progress of the disease and preferable feather of oxysterols as potential biomarker in schizophrenia.

To examine whether oxysterols levels were associated with psychiatric symptoms, we then investigated the correlation between circulating 24OHC and 27OHC and positive and negative symptoms scales (PANSS) scores in the schizophrenic patients. First, we found that 24OHC levels positively correlated with total PANSS and negative symptom scores (Tables 2, 3). As accumulating evidence revealed the negative symptoms were related with disturbance in serotonin and glutamate transmission (97–99), which can be regulated by 24OHC-induced disequilibrium of cholesterol homeostasis in the brain through lipid raft microdomains on the cell membranes (100, 101), we hypothesize that the correlation of 24OHC to negative symptoms in schizophrenia might be mediated through serotonin signaling. In addition, 24OHC as an allosteric regulator of NMDA receptor can increase the glutamatergic signaling transmission which also aggravates the excitotoxic injury in schizophrenia (20–22, 102). On the other hand, our present study also showed an inverse correlation between 27OHC levels and positive symptom scores in schizophrenia patients (Tables 2, 3). As positive symptoms were associated with increased hyperdopaminergic activity in schizophrenia (103, 104), 27OHC as a selective estrogen receptor modulator can attenuate dopamine level by reducing the expression of tyrosine hydroxylase (TH, the rate-limiting enzyme in dopamine synthesis) *via* the inhibition of estrogen signaling (105–107).

However, whether oxysterols could be potential biomarkers for schizophrenia still needs more advanced investigation. For example, a recent study from Chiappelli et al. based a large sample (226 schizophrenia patients and 204 controls) found no significant difference in plasma 24OHC levels between patients and controls (32). Furthermore, they also found no relation between plasma 24OHC levels and whole-brain white matter average fractional anisotropy or cortical thickness (32). While the report from this study is not consistent with our hypothesis that the elevation of 24OHC was associated with brain structure, it is important to notice that the change of plasma 24OHC levels in the psychiatric disease might have brain regional specificity. For example, a postmortem study of suicide samples (mainly from MDD patients) showed elevated 24OHC levels in the prefrontal cortex (28), while another report indicated no significant change in 24OHC levels was found in association cortex (108). The lack of assessment of sub-brain structural abnormalities in schizophrenia patients left open the question whether the elevation of 24OHC was associated with regional brain structure.

Several limitations were found in this study. First, 30% schizophrenia patients were antipsychotic-treated individuals. Although we found no effect of antipsychotic treatment on 27OHC levels (Supplementary Figure 1), we could not exclude the possible confounding effect of medications on cognitive function and clinical symptoms in our study. Second, the plasma total cholesterol levels were not detected in this study. Third, the lack of the follow-up of CHR participants left open the question if plasma oxysterol levels changed with the progress and outcome of schizophrenia.

Taken together, our study indicated the elevation of circulating 24OHC levels and the reduction of 27OHC levels in schizophrenia patients, and this alteration were related to the severity of psychiatric symptoms in schizophrenia. This suggested that plasma oxysterols might be the potential biomarker in schizophrenia.

## Data Availability Statement

The raw data supporting the conclusions of this article will be made available by the authors, without undue reservation.

## Ethics Statement

The studies involving human participants were reviewed and approved by Independent Ethics Committee (IEC) of the Beijing Anding Hospital, Capital Medical University, China. Written informed consent to participate in this study was provided by the participants' legal guardian/next of kin.

## Author Contributions

CW, RL, and TJ obtained funding for this study. ZS, RL, and CW designed the research. ZS, LZ, QB, ZM, YH, and YL performed the experiments and statistical analysis. ZS and RL wrote the manuscript. All authors contributed to the article and approved the submitted version.

## Conflict of Interest

The authors declare that the research was conducted in the absence of any commercial or financial relationships that could be construed as a potential conflict of interest.

## Publisher's Note

All claims expressed in this article are solely those of the authors and do not necessarily represent those of their affiliated organizations, or those of the publisher, the editors and the reviewers. Any product that may be evaluated in this article, or claim that may be made by its manufacturer, is not guaranteed or endorsed by the publisher.

## Supplementary Material

Supplementary Figure 1 Differences in plasma oxysterol levels between antipsychotic-naive and antipsychotic-treated patients with schizophrenia. The plasma 24OHC levels and 27OHC levels in antipsychotic-naive or antipsychotic-treated patients with schizophrenia are shown in **(A,B)**, respectively. No significant difference was found between these two groups in patients.
