# Supplementary figures and images for: Brain-Specific Oxysterols and Risk of Schizophrenia in Clinical High-Risk Subjects and Patients With Schizophrenia
Source: Front Psychiatry. 2021 Aug 2;12:711734. doi: 10.3389/fpsyt.2021.711734 (PMC8367079; doi:10.3389/fpsyt.2021.711734)

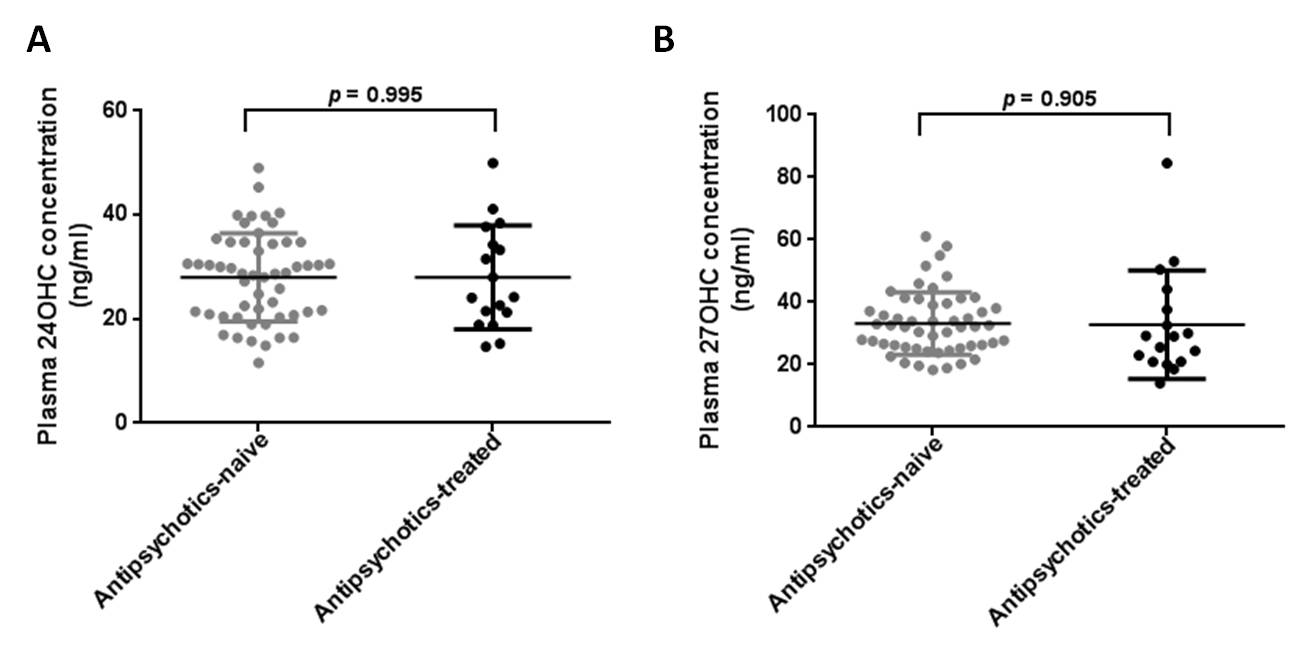

Supplement: Supplementary Figure 1 — Differences in plasma oxysterol levels between antipsychotic-naive and antipsychotic-treated patients with schizophrenia. The plasma 24OHC levels and 27OHC levels in antipsychotic-naive or antipsychotic-treated patients with schizophrenia are shown in (A,B), respectively. No significant difference was found between these two groups in patients. [file Image_1.JPEG]
